# Quantifying the Impact and Relevance of Scientific Research

**DOI:** 10.1371/journal.pone.0027537

**Published:** 2011-11-16

**Authors:** William J. Sutherland, David Goulson, Simon G. Potts, Lynn V. Dicks

**Affiliations:** 1 Conservation Science Group, Department of Zoology, University of Cambridge, Cambridge, United Kingdom; 2 Biological & Environmental Sciences, School of Natural Sciences, University of Stirling, Stirling, United Kingdom; 3 Centre for Agri-Environmental Research, School of Agriculture, Policy and Development, University of Reading, Reading, United Kingdom; Canadian Agency for Drugs and Technologies in Health, Canada

## Abstract

Qualitative and quantitative methods are being developed to measure the impacts of research on society, but they suffer from serious drawbacks associated with linking a piece of research to its subsequent impacts. We have developed a method to derive impact scores for individual research publications according to their contribution to answering questions of quantified importance to end users of research. To demonstrate the approach, here we evaluate the impacts of research into means of conserving wild bee populations in the UK. For published papers, there is a weak positive correlation between our impact score and the impact factor of the journal. The process identifies publications that provide high quality evidence relating to issues of strong concern. It can also be used to set future research agendas.

## Introduction

Research is paid for by taxpayers, organisations and individuals because of the benefits to society. These benefits might be economic if the research generates commercial opportunities. They might be improvements to quality of life or sustainability. In the case of curiosity-driven research, enhancing the extent of human knowledge is itself a benefit. There have recently been calls for greater quantification of the impact of scientific research on society. This is a necessary first step towards evaluating returns on research investment, or the effectiveness of the research effort at providing societal benefit. In a number of countries, research funding bodies have initiated efforts to assess research impact, including the United Kingdom, the United States, the Netherlands and Australia [1], [2].

The impact of research can be assessed qualitatively or quantitatively. Qualitative approaches, such as the one recently trialled by the UK government's Higher Education Funding Council, involve expert panels evaluating impact, for example as high, medium or low, based on written descriptions of impact [1]. Quantitative approaches can involve numerical indicators derived from scoring systems or questionnaires focused on the various possible impacts of a research programme or project. The approach developed in the UK for the Arthritis Research Campaign by Wooding *et al*. [3], [4] is largely quantitative, and measures the impact of a funding body's research portfolio based on self-reported impacts. The STAR METRICS system in the United States [2] aims to capture data on scientific outputs and activities linked to research investments systematically. This will enable quantitative assessment and analysis of the impacts of research. It is expected to take at least five years.

**Figure 1:**
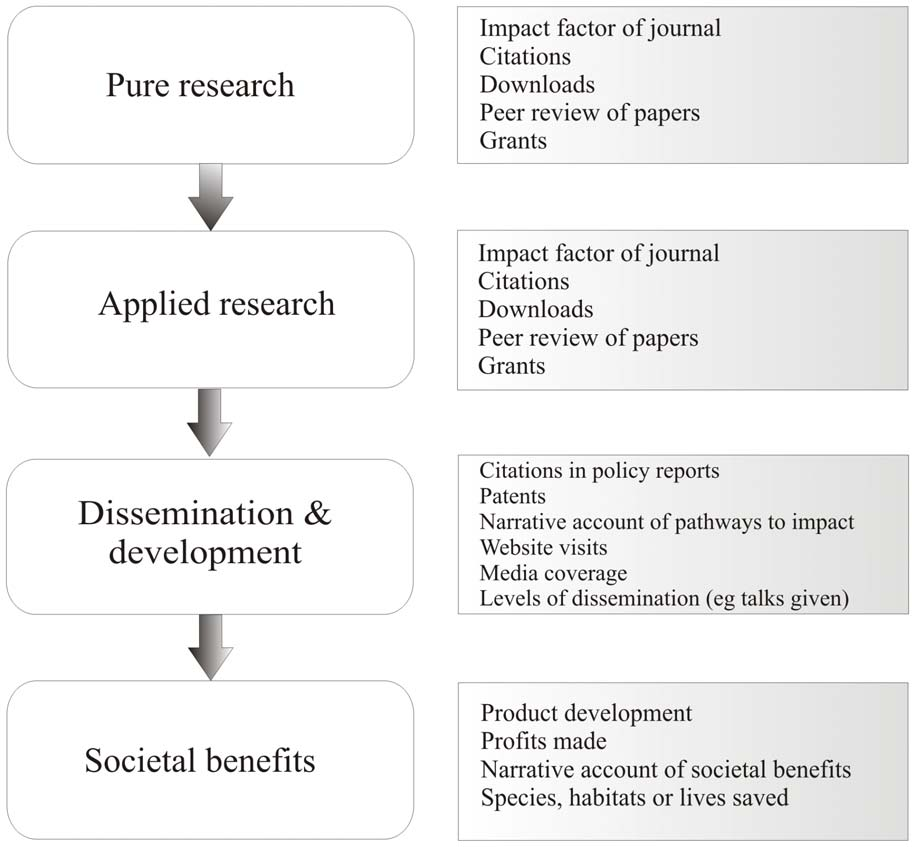
Main components of the progression of science into practice and societal benefits (left), with some existing measures of impact and quality (right).

Fig. 1 uses the linear model of innovation to illustrate how the quality and impacts of research can be assessed at different points in the development of research into policy and practice. The linear model has long been used to justify the funding of basic research [5], although it is rightly criticised as simplistic. It shows how pure research could lead to societal benefits, but it does not, for example, allow for crucial feedback processes through which societal needs shape pure and applied research. Nonetheless, we find the linear model in its simplest form provides a useful basis for discussing the different approaches to measuring the impacts of research.

There are several problems associated with following research impacts through the different stages of applied research and dissemination and development shown by the linear model, whether using qualitative or quantitative approaches. These problems are reviewed by Frank and Nason [6], and we summarise them and extend the taxonomy below.

### Attribution

Attributing societal impacts to a particular piece of research can be challenging, as seen in identifying key stages in the development of streptomycin [7] or in the acrimonious squabble over the discovery of insulin [8].

### Counterfactual

Could the claimed impact have taken place without the research? While this is obviously impossible for technological developments entirely dependent upon a research development, such as DNA fingerprinting, in other areas decisions could be made for other reasons. For example, the United Kingdom's greenhouse gas emissions fell in the 1990s and 2000s, following its acceptance of research showing the relationship between greenhouse gases and climate change. However, a large measure of this reduction was due to de-industrialisation, and a switch from coal- to gas-fired electricity generation, which would have occurred anyway [9].

### Time lag

The gap between discovery and application can be considerable, as illustrated by the gap of up to 17 years (median 6.4 years) between the registration of a new drug and its approval by the Food and Drug Administration in the US [10]. Similarly, the mean time lag between spending on cardiovascular disease research and the benefits to society through measurable health gains was estimated at ranging from ten to twenty-five years with a mid point of seventeen years [11].

### Factors beyond science

Whether a discovery has an impact on society is not just dependent on the quality or relevance of the underlying science. The extent to which research outcomes are used may depend on politics, as seen in climate change science [12], or a combination of commercial factors such as marketing, usability and pricing, as in the development of videocassette formats [13]. Research that identifies problems that are not acted upon, or provides practical solutions that are not adopted for reasons such as those listed here, would be accredited with no impact by some methods of evaluation. However, such research still deserves credit for its potential impact.

### Complex nature of impact

Discoveries with commercial applications have obvious financial impacts. Medical discoveries sometimes have impacts quantifiable in terms of lives saved. But much of science has impacts that are difficult to define, particularly when the benefits are related to quality of life or some other dimension of societal development [14]. Finding an appropriate metric with clear links to the research is a huge challenge.

The UK government accepts that it is impractical to measure the impact of recent research through its direct benefits to society. This has led to the decision to use ‘pathways to impact’ – a qualitative assessment of the attempts of researchers to ensure their results are applied, through knowledge transfer activities such as the development of websites and activities to engage the public and stakeholders [15].

These knowledge transfer activities are important and seem likely to have benefits. If there is good engagement between scientists and end users of research at every stage in the research process, it should reduce the likelihood of research not achieving its potential impact due to unforeseen societal factors.

However, the amount of active communication that occurs is not a reliable indicator of the relevance, or usefulness, of a given piece of research to society, or of its contribution to understanding in that area. It is therefore unreliable as an estimator of actual impact. Individual promotion of certain pieces of evidence could even be counterproductive. For example, in a recent review of the effectiveness of methods to stop smoking, Chapman and Mackenzie [16] argue that the promotion of research on medical methods such as nicotine replacement therapies has led to an overemphasis on ‘assisted cessation’, despite good evidence that the most successful method is to stop smoking unassisted. Research into medical methods to stop smoking appears to have achieved more impact than it deserves.

We propose a quantitative approach in which impact scores for individual research publications are derived according to their contribution to answering questions of relevance to research end users. It builds upon a developing framework of literature assessment to support evidence-based policy and practice in biodiversity conservation [17], [18]. To demonstrate the approach, here we apply it to evaluating the impact of research into means of restoring and enhancing wild bee populations in the UK – a topic of considerable interest due to concern over the decline in wild pollinators [19].

## Methods

### Ethics statement

The Cambridge Psychology Research Ethics Committee has given ethical approval to this research project and each practitioner who took part gave us their informed written consent.

We identified 54 interventions that could benefit wild bee populations in the UK, based on our own knowledge, the literature and advice from an international seventeen-member advisory board (these advisers are named in [20]). The list of interventions, given in Table S1, is organised into categories based on the International Union for the Conservation of Nature (IUCN) classifications of direct threats and conservation actions. We used non-judgemental words to describe the categories of intervention for this exercise, to avoid bias, choosing awareness over education, for example, and agricultural chemicals over pollution.

We searched the literature for publications that test the effectiveness of any intervention on the list. The methods and results of this review are published as a ‘synopsis’ of evidence on bee conservation [20].

In total, 159 individual publications are included in this exercise. They include 149 published scientific papers, 4 reports, 3 books or book chapters and 3 PhD theses.

The five year Journal Citation Report (JCR) impact factor was obtained for each publication that was in a scientific journal. For relatively new journals, where a five-year impact factor was not available, the impact factor for the most recent year (2009) was used instead. We compare our impact score with the journal impact factor, rather than using a specific metric for individual publications, such as the number of citations, because publication-specific measures are very time dependent. Many of the publications are very recent (2009 or 2010) and have not had time to accumulate citations. The JCR impact factor is widely used by scientists and funders to assess the quality of publications.

We provided the list of interventions to a group of people who use research on bee conservation. They should be considered a consulted group of conservation practitioners and advisers (referred to here as ‘practitioners’). We did not attempt to sample the full population of people with an interest in bee conservation.

We used purposive sampling (subjective sampling with a purpose) as described by Sutherland *et al.* [21] to invite a diverse set of suitable practitioners. Our sample was stratified to represent as much of the UK as possible, and to represent what we consider to be the important interest groups in the policy and practice of bee conservation - national and local policymakers, conservation NGOs (non-governmental organisations), farmers, farm adviser and consultants, and researchers.

We initially approached 113 practitioners. They comprised ecological consultants with an interest in insect conservation identified from the Institute of Ecology and Environmental Management online members database, representatives from key UK conservation agencies and Government environment departments (Natural England, the Northern Ireland Department of Environment The Rural and Environment Directorate of the Scottish Government, the Countryside Council for Wales, the Department for Environment Food and Rural Affairs), representatives from UK NGOs with an interest in insect conservation (including Buglife, Butterfly Conservation, The Bumblebee Conservation Trust and the Bees, Wasps and Ants Recording Society), researchers working on issues related to bee conservation and members of the Association of Local Government Ecologists (one from each county was approached, selected at random from the online database of members). In thirty cases, our initial contact suggested someone else with more appropriate experience and knowledge. The final group of respondents comprised 8 national policymakers, 13 local/regional government ecologists, 9 from conservation NGOs, 6 academics and 8 farmers/farm advisers/farm consultants −44 respondents in total (of 143 approached, giving a response rate of 31%).

These people were asked to allocate 1,000 points between the different interventions, according to how they consider each action should be prioritised. They were also asked to ignore prior knowledge of effectiveness. This was an attempt to avoid bias against interventions which science has found are not very effective. An early study that found a negative result, such as Fussell and Corbet's 1992 trial of bumblebee nest boxes [22] that found very low uptake rates for the boxes (average 1.5%), may have already exerted its impact on policy and practice, resulting in the intervention being given a low priority score. To reflect the impact of such studies in an unbiased way, you would need to have generated a priority score from practitioners before any scientific knowledge was available. This is not usually possible.

The order in which interventions were presented was varied to enable us to test whether order affected scoring. Four different score sheets were used, in which the categories were presented in a different order. The re-ordering was done systematically, by reversing the order or switching the middle categories to the outside for both the original and reversed order, so that each intervention appeared in a range of positions, near the beginning, near the end or somewhere in the middle of the list.

For each intervention a priority score was generated by taking a mean score across all practitioners.

Three experts in bee ecology and conservation (LVD, DG and SGP) assessed the evidence for each intervention, and the contribution and relevance of each publication. They generated scores using the Delphi technique [23]. The experts initially scored independently and all the scores were shown to all three experts. Each intervention and each piece of evidence (publication) was then discussed at a one day workshop, chaired by WJS, during which the experts independently adjusted their scores. A mean score across the three experts was used as the final score for each intervention or publication.

Certainty of knowledge about the effectiveness of each intervention in benefitting wild bee populations was scored on a percentage scale (0% = no useful evidence presented, 100% = fully resolved).

The percentage contribution of each publication to knowledge was assessed for each intervention, starting with the oldest paper and considering additional advances provided by each subsequent paper. Scores were adjusted for study design and additional advances. A solitary publication scored 100%. Papers showing negative results were considered as contributions to knowledge either by showing that an intervention does not work or by showing that the response can be variable. Reviews were scored for any additional contribution they provided.

Papers including additional research unrelated to UK bee conservation should be credited for that. To achieve this, the percentage relevance of each individual publication to UK bee conservation was assessed by evaluating the proportion of the study that tested conservation interventions for UK bees. We make the assumption that additional equivalent work outside bee conservation has equal impact. A study researching bees and butterflies equally was given a relevance score of 50% and thus assumed to have double the total impact of an equivalent study just on bees (following Eqn. 2).

We adopted the same approach for field research carried out outside the UK. We assessed the relevance of the work to answering the questions in the UK. Thus if the research involved UK species, or was in habitat very similar to those in the UK (such as in the Netherlands), then the relevance was high. If the work was on species with no close relative in the UK then the score was lower. The precise value attributed to relevance was a matter of expert judgement.

Scoring was carried out to avoid prejudice against non-UK work, by matching reduced scores for certainty of knowledge and contribution with similarly reduced relevance. A paper on a very different community would be given a reduced contribution - say half what it would have been given if the same research had been done in the UK, because the findings are of limited use to the UK situation. If the paper provided the only evidence for a given intervention (100% contribution), the certainty of knowledge score would be halved. Either way, the impact score would be reduced by half. To counteract this in our assessment of total impact, the paper would be given a similarly reduced relevance score of say, 50%, doubling the total impact to reflect its importance outside the UK.

Impact scores for each publication were generated as follows:
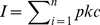
(1)

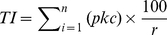
(2)Where *I* = impact score (bee conservation), *TI* = total impact score, *p* = priority score, *k* = certainty of knowledge score, *c* = contribution to knowledge, *r* = relevance and *n* is the number of interventions for which a given publication provides evidence.

### Statistical analysis

To test whether the order of presentation of interventions biased the scores, we ran a Principal Components Analysis on the scorers' results (44 scorers, 54 variables for each), using a correlation matrix so the variables were standardised and therefore given equal weight. We used analysis of variance on the first two principal component axes scores to test for any difference between scorers according to the scoresheet they used.

By the same method we tested for any significant difference between the five groups of scorer: national policymakers, local/regional government ecologists, non-governmental conservation organisations, academics and farmers/farm advisers/consultants.

To test for a correlation between the journal impact factor and our impact scores, we used Spearman's rank correlation test, using an asymptotic formula that allows for ties. This test was chosen because the JCR impact factors (n = 135) did not meet the assumption of normality, even after transformation.

To test for a correlation between the certainty of knowledge score and the number of publications for each intervention, we also used Spearman's rank correlation test, because the number of publications per intervention was not normally distributed, even after transformation.

## Results

**Figure 2:**
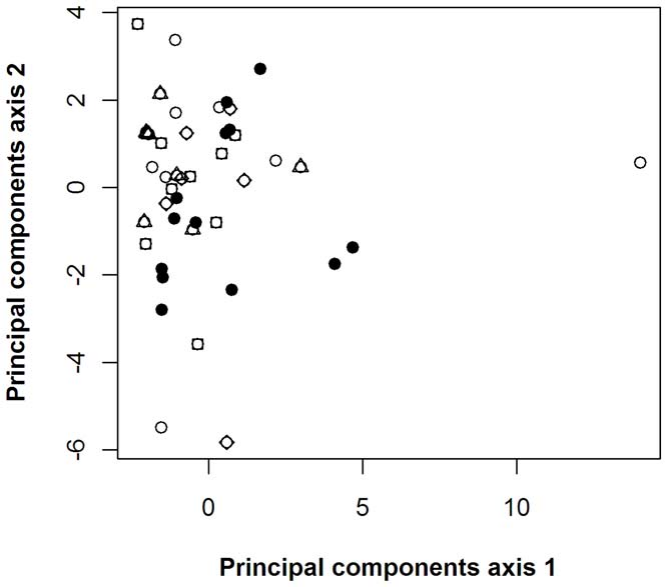
Plot of the first two principal components axis scores for each scorer. Here scorers are identified according to group: national policymakers = open circles, local/regional government ecologists = closed circles, representatives of non-governmental conservation organisations = squares, academics = diamonds, farmers/farm advisers/farm consultants = triangles. Analyses of variance of the first two principal components axis scores for each scorer showed no significant difference between different scoresheets (p = 0.636 for axis 1; p = 0.364 for axis 2) or between the five scorer groups (p = 0.085 for axis 1, p = 0.705 for axis 2). A single scorer in the national policymaker group scored differently from others, giving relatively high scores for the interventions in the ‘*Ex situ* conservation’ category. With this single scorer removed from the analysis, the p value in the analysis of axis 1 scores by scorer group was 0.2888.

Our Principal Components Analysis of the 44 practitioners who provided priority scores did not group them into discernible groups. Scoring was not significantly different according to the order in which interventions were presented, nor between different groups of scorers (see fig. 2).

**Figure 3:**
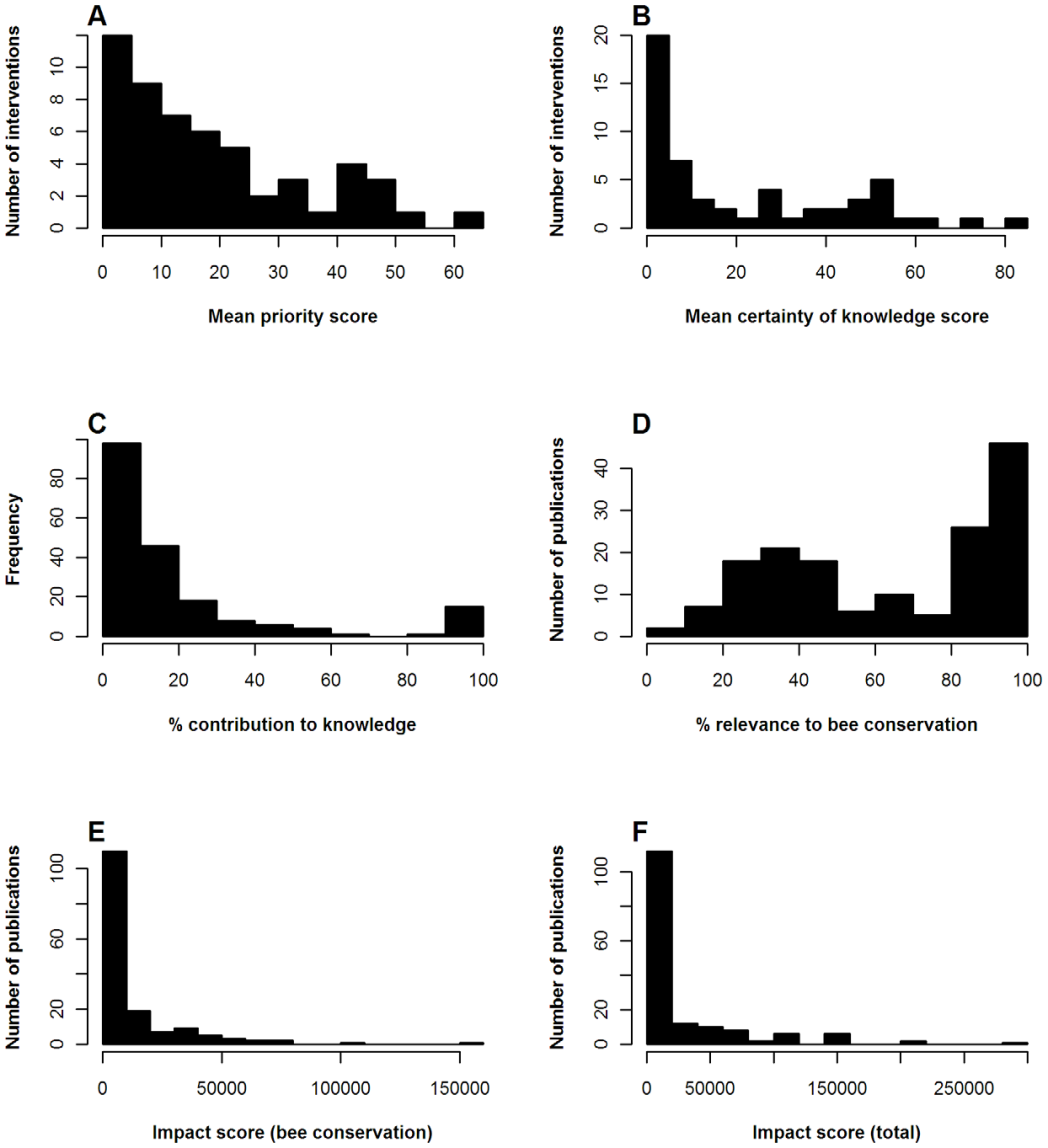
Frequency histograms of impact scores and their components. **A–B** Scores for each of the 54 interventions. **A** Priority scores provided by practitioners, **B** certainty of knowledge scores provided by expert group; **C** contributions to knowledge - each publication may have one or more of these, depending on how many interventions it relates to, N = 197; **D** relevance scores and **E–F** impact scores for each publication, N = 159. Relevance scores provided by expert group. Impact (bee conservation) scores are calculated according to Equation 1, without use of relevance scores. Impact (total) scores are calculated according to Equation 2.

Fig. 3 shows the distribution of results for impact scores and their components. Fig. 3A shows the distribution of priority scores assigned to each intervention by the practitioners (mean priority score across interventions = 18.5, range 1.4–62.3). Nine of the ten highest scoring interventions for priority are shown in Table 1. The only intervention in the top ten priority scores not shown here was ‘Sow uncropped arable field margins with a native wild flower seed mix’, which ranked 8^th^, with a priority score of 41.4.

**Table 1:** Research priorities identified.

Intervention	Certainty of knowledge	Mean priority score
Increase the proportion of natural habitat in the farmed landscape	0.0	62.3
Restore species-rich grassland vegetation	36.7	51.4
Protect existing natural or semi-natural habitat to prevent conversion to agriculture	0.0	46.8
Connect areas of natural habitat together	0.0	46.3
Introduce agri-environment schemes generally	30.0	45.9
Provide training to conservationists and land managers on bee ecology and conservation	0.0	44.4
Increase the diversity of nectar and pollen plants in the landscape	6.0	41.9
Restore species-rich grassland on road verges	13.3	40.8
Plant parks and gardens with appropriate flowers	46.7	37.6
Raise awareness amongst the general public through campaigns and public information	0.0	32.5

Ten interventions for wild bee conservation with high priority scores (>31.9, above the 80% quantile) and low certainty of knowledge (<47.3, below the 80% quantile).

The certainty of knowledge (3B) scores assigned to each intervention by our expert group were typically under 20% (mean certainty of knowledge score = 21.7%, range 0–81.7%). For most interventions it is far from clear how effective they are. Almost all publications looked at the local effect of the intervention (such as higher bee density on field edges following flower planting) but not at the effect on bee populations. As the objective is to conserve wild bee populations, none of the certainty of knowledge scores approached 100%. The highest scoring intervention was ‘Provide artificial nest sites for solitary bees’, about which there were 33 publications, including four that assessed the impacts of nest boxes on bee reproductive success or population numbers.

The contribution score (3C) substantially depends upon the number of publications related to each intervention (mean contribution = 20.3%, range 1–100%). With 10 contributing papers the mean contribution will be 10%, with each adjusted according to publication sequence and quality. Fourteen interventions had single pieces of evidence whose contributions to knowledge were therefore 100%.

For some interventions, contribution scores were relatively evenly distributed across a number of publications. For example, for the intervention ‘Sow uncropped arable field margins with a native wild flower seed mix’ we identified seven publications, all describing work on UK farmland and published between 1999 and 2007. Their contribution scores ranged from 11.7 to 18.7. The highest scoring publication here was not the earliest, but the most extensive - a replicated controlled trial across thirty-two 10 km grid squares in England [24].

For other interventions, the bulk of knowledge was assessed to have come from a small number, or just a single publication. For example, the intervention ‘Eradicate threatening non-native bees or bee parasites’ had two associated publications. One was a small trial of a method for killing individual honey bee *Apis mellifera* colonies at a site in the USA, using insecticide-laced syrup [25]. This was given a low contribution score of 1.3%. The other was a replicated controlled trial of the efficacy of removing non-native bumblebees (*Bombus terrestris*) at six sites in Japan over two years [26]. This was felt to have contributed most of the knowledge and given a contribution score of 98.7%. The certainty of knowledge score for this intervention was 8.3%, reflecting the fact that evidence was only available for two particular species in two specific locations.

Relevance (3D) has two peaks: 46 publications focussed on bee conservation techniques, carried out in the UK or western Europe, or in controlled environments, have very high (>90%) relevance. For example, Pywell *et al.*'s study of agri-environment scheme options for bumblebees [24] scored 100% for relevance. Those at 20–50% examined a range of taxa or issues not included in our list of interventions or were carried out further away from the UK. For example, a paper looking at the effects of management to restore heathland on several insect groups, one of which was bumblebees [27], was given a relevance of 26.7%, because only a proportion of its results were relevant to bee conservation. A paper that monitored bees visiting an urban garden planted with bee-friendly flowers in California, USA [28] was given a relevance score of 61%, because it was in a habitat very different from the UK, with a very different bee fauna. (Overall mean relevance score = 64.5%, range 6.3–100%).

The impact scores for individual publications (3E) show a strong positive skew with most papers having relatively low impact. The total impact, obtained by including publication relevance and so allowing for impacts outside UK bee conservation (3F), has an even greater skew (mean impact score = 12,069, range 115.9–152,732; mean total impact score = 26,830, range 115.9–289,952).

**Figure 4:**
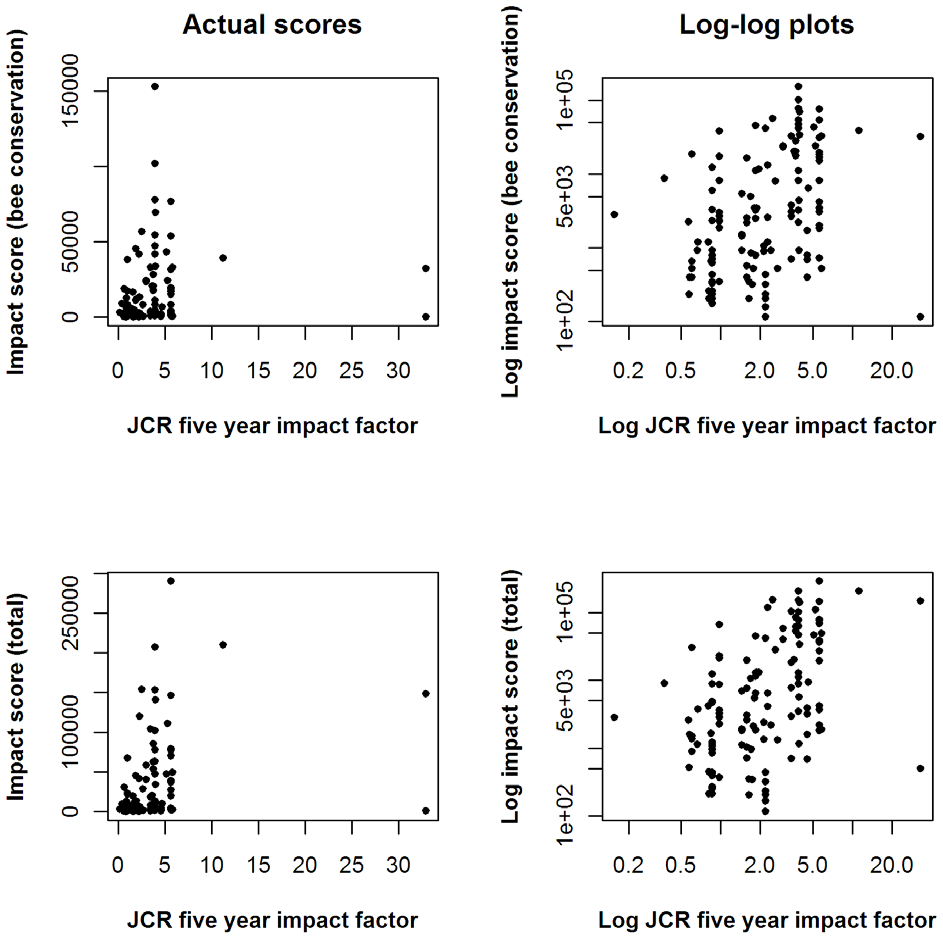
Impacts in bee conservation (top), and estimated total impacts (bottom), related to impact factor of publishing journal. These graphs include 135 publications published in journals for which impact factors are available. The log plots are presented to make the weak correlation easier to see. The publications in high impact journals with low impact scores are discussed in the text.

The scoring identifies a number of publications with particularly high impact. The two highest scoring publications for impacts in bee conservation (see fig. 4, top left) each contain evidence relating to four different interventions. Both are replicated controlled trials of the use of farmland managed under different agri-environment scheme options by bumblebees, in England [24] or Scotland [29]. In both cases, two of the interventions tested have priority scores higher than the 80% quantile (‘Restore species-rich grassland vegetation’ [priority score = 51.4] and ‘Sow uncropped arable field margins with a native wild flower seed mix’ [priority score = 41.4], for example).

Both the high scoring publications are considered the largest contributors to certainty of knowledge for three of the four interventions they assess, either because they were well designed, extensive experimental studies, or because they were the first and only publication to directly address the question. Lye *et al.* [29] is the only publication to provide evidence for the effects of two interventions: ‘Manage hedges to benefit bees’ and ‘Increase areas of rough grassland for bumblebee nesting’.

For total impact score, there are three particularly high scoring publications (fig. 4, bottom left) [30], [31], [32]. These three papers all provide evidence relating to one or more interventions that achieve priority scores higher than the 80% quantile. They also have relatively low relevance to bee conservation (relevance scores from 6–20%), because they consider four or more other species groups apart from bees, so their impacts are multiplied. For example, Meek *et al.* [32] consider the effects of three different bee conservation interventions on five different species groups: butterflies, spiders, ground beetles and plants as well as bumblebees. This makes the assumption that the impact of a publication on other taxa is similar to that on bees, an assumption which could be tested if our method were applied for each taxon.

Fig. 4 shows the relationship between the impact of a paper assessed by this method and the impact factor of the journal in which it was published. A significant, but weak, positive correlation exists between the journal impact factor and our impact score. The correlation with journal impact factor is slightly stronger when impacts outside bee conservation are taken into account. (Spearman's rank correlation coefficient r_s_ = 0.457, p = 2.48×10^−9^ for impact score (bees only), and r_s_ = 0.491, p = 1.41×10^−9^ for total impact). Papers in the higher impact journals (>5) that receive relatively low ‘total impact’ scores tend to be recent publications relating to interventions given low priority scores by practitioners and policymakers and for which there are already many good papers.

One of the two publications that are in very high impact journals has particularly low impact scores. This particular study [33] reared bumblebee colonies from wild-caught queens in laboratory conditions. It was one of 27 publications providing evidence for the intervention ‘Rear declining bumblebees in captivity’, which had a very low priority score of 1.68. The publication therefore scored low for both priority and contribution to knowledge. However, the publication demonstrated reproduction by worker bumblebees in colonies other than their own, a very important finding in the theoretical field of evolutionary biology. The test of rearing bumblebees was supplementary to its primary focus. To reflect this, the publication was given a low relevance score by our process (23.3%), but its total impact score was constrained by components of the score (priority and contribution to knowledge) for which values were defined in the context of bee conservation. This case serves to illustrate a potential shortcoming of our method, when attempting to estimate the total impact of publications that address problems in very different areas, or combine ‘pure’ research with the application of methods relevant to policy and practice in a different area.

**Figure 5:**
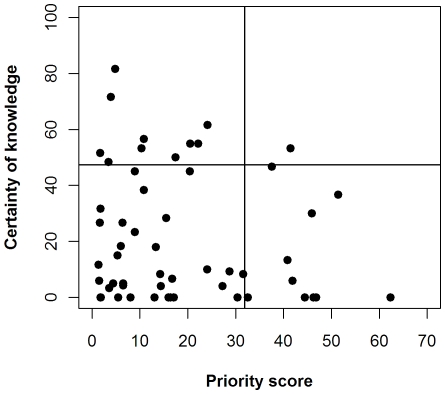
A method for setting research priorities. Each intervention to conserve wild bees is plotted according to its mean certainty of knowledge score (assessed by three experts) and mean priority score (assessed by 44 practitioners). The ten interventions in the ‘research priority’ quadrant of high priority but low certainty of knowledge (bottom right) are listed in table S2. Lines are drawn at the 80% quantiles for knowledge and priority scores.

Our approach can be used to derive research agendas. Fig. 5 shows the interventions plotted by certainty of knowledge (the extent to which the issue is solved) and priority to practitioners. The interventions that are largely unsolved but assigned high priorities, towards the bottom right, can be considered research priorities. These are listed in Table 1.

**Figure 6:**
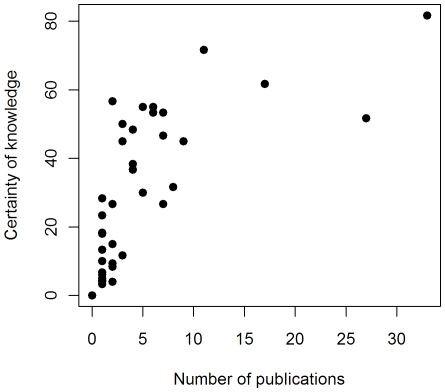
Certainty of knowledge score related to the number of publications addressing effectiveness for each intervention.

Figure 6 shows that the certainty of knowledge score is positively correlated with the number of publications that address effectiveness for each intervention (Spearman rank correlation coefficient r_s_ = 0.914; p = 2.2×10^−16^). The data suggest an asymptotic relationship, in which acquisition of knowledge is greatest in the first few publications, followed by diminishing returns on research investment as the number of publications increases.

## Discussion

We have shown how it is possible to measure the impact of research publications within a clearly defined policy objective - the conservation of wild bees. This is quite different to previously discussed quantitative approaches to measuring research impact. Rather than taking a research programme, project or publication as a starting point and asking what its contributions to society have been, our approach takes the issues society wants answered as a starting point and asks how much each piece of research has contributed to answering them.

The research we have included almost entirely falls into the ‘applied research’ box in fig. 1. Our method is most appropriate to this kind of research because it requires an agreed set of possible solutions or questions. It is feasible to generate these in areas where there are clear problems, such as the conservation of biodiversity, climate change, sustainable development or health. A number of studies have identified questions of highest priority to policy, for example, in global conservation [34], US environment [35] and global agriculture [36]. Feasible options or interventions are being listed in other policy areas [37], [38], and in some cases also evaluated by multi-sector stakeholder groups [39], [40], [41].

Whilst in theory it is possible to identify priority questions for more theoretical subjects, such as particle physics or theoretical ecology, and then assess the importance of individual research publications in providing relevant evidence, this is likely to be too subjective to be useful. It is also likely to miss important impacts, because knowledge emerging from pure theoretical research can have unexpected uses. For example, the researcher employed to search for ciphers in Shakespeare's writing subsequently used that knowledge to crack the Japanese machine cipher in the Second World War [42].

Our measure is unaffected by the problems we identified in other methods of measuring impacts, because it does not try to track the impacts of a piece of research as they travel through to societal benefits (downwards in fig. 1). It is not necessary to define or account for all the possible ways a piece of research can be demonstrated to have exerted its impact, such as through improved quality of life, new commercial ventures or attributable changes in policy. Instead we begin with societal needs (strictly, the needs of the most interested stakeholders in a clearly defined area) and track upwards in fig. 1. We assess research according to the importance of the question tackled and the quality of the research. Research that enhances knowledge and contributes to decision-making is valued by this process even if it is not finally chosen to provide a solution. Our priority scoring by end users takes into account logistical issues associated with the development stage because their scores prioritise actions they are already implementing or which they consider to be feasible.

Our method allows researchers and funding bodies to evaluate the impact of research in a given policy area and gives a quantitative indication of the potential for impact in other policy areas.

We do not suggest that our approach becomes a standard means of assessing impact across the science budget. It has three main problems.

Firstly, the benefits to society of scientific discoveries cannot always be measured purely in terms of their application, or contribution to solving specific problems. This does not mean that pure research is entirely excluded from our process. Much research that has societal impact combines practical application with purely theoretical questions. The theoretical aspects may be undervalued by our method if they are in an unrelated area, as in the study of bumblebee breeding behaviour discussed above. However, our method can easily evaluate interdisciplinary research, or combinations of pure and applied research, providing the disciplines have come together to focus on developing solutions to a particular societal problem. There are calls for an increase in this kind of integrated, problem-focussed interdisciplinary research in the context of environmental change [43], [44].

A second problem is that the impact score depends to some extent on who you ask to set the priority scores, a process that involves subjective sampling. We have accounted for this by purposive sampling that draws on the important interest groups. However, if you changed the set of practitioners, the outcome might be different. For example, in this exercise, two interventions that deal specifically with the threat of pesticides - ‘Reduce pesticide or herbicide use generally’ and ‘Restrict certain pesticides’ - did not fall in the list of top ten priorities (by priority score) or the list of research priorities (given in Table 1). These two interventions ranked 15^th^ and 17^th^ of 54 by priority score, reducing the impact scores of the publications that provided relevant evidence. Had we approached a different selection of conservation NGOs, or opted for a higher proportion of NGO representatives in our sample, these interventions might have been more prominent. If there were very strong differences of opinion between interest groups, as we have shown is not the case here, it would be possible to compile impact scores using the priority scores from each interest group separately and compare the outcomes.

A potential bias is introduced by the practitioners' prior knowledge. Although we asked them to ignore their prior knowledge of the effectiveness of interventions, we admit that this is an almost impossible task, given that we selected people with an interest in bee conservation. As discussed in the Methods section above, this could introduce a bias in favour of publications with a positive outcome, and against publications with a negative result, although negative results that prevent resources being wasted are at least as important to society.

There are two possible ways to evaluate the extent of this bias. One is to gauge the level of knowledge amongst practitioners at the same time as gathering their priority scores, perhaps by asking ‘In your opinion, does this intervention work?’, or ‘How much scientific evidence do you think there is about whether this intervention works or not?’. It would then be possible to identify interventions for which practitioners may have been biased by prior knowledge. Another approach would be to identify interventions for which the evidence provides a clear message, and ask practitioners whether their scores for these would be different in the face of new conflicting evidence. In the example of bumblebee nest boxes given above, it is very possible that the low priority score given to this intervention (priority score 3.91, ranked 45^th^ of 54 interventions) would remain low even if bumblebee nest boxes were shown to be very effective in the UK, because of the cost and practical difficulties of using them on a large scale.

The third potential drawback of our method is that it is time consuming to carry out as it requires a thorough literature review and gathering of scores for both research publications and interventions or solutions. If wishing to assess a particular paper with accuracy it is important that the review is comprehensive.

There is a mounting effort to compile scientific evidence for particular interventions in a way that is accessible to policymakers and practitioners [17]. With interventions already evaluated [39], [40], [41] and evidence already compiled, assessing the impact of individual publications using our method requires only a small expert committee to assess the certainty of knowledge, contribution and relevance of each publication. This approach could thus readily be applied to fields such as medicine and climate change where there is existing extensive synthesis of the literature.

## Supporting Information

Table S1 **The full list of interventions to benefit wild bee populations, as presented to practitioners.**
(DOC)
