# Supplementary material for: Quantifying the Impact and Relevance of Scientific Research
Source: PLoS One. 2011 Nov 16;6(11):e27537. doi: 10.1371/journal.pone.0027537 (PMC3217965; doi:10.1371/journal.pone.0027537)
Supplement: Table S1 — The full list of interventions to benefit wild bee populations, as presented to practitioners. (DOC) [file pone.0027537.s001.doc]

**Supporting information for Sutherland *et al* (2011) Quantifying the impact and relevance of scientific research**

**Table S1 The full list of interventions** to benefit wild bee populations, as presented to practitioners.

| **Threat or action category** | **Intervention** |
| --- | --- |
| **Urban land use** | Plant parks and gardens with appropriate flowers |
|  | Practise/encourage 'wildlife gardening' |
|  | Protect brownfield sites |
|  | Conserve old buildings or structures as nesting sites for bees |
| **Agricultural land use** | Protect existing natural or semi-natural habitat to prevent conversion to agriculture |
|  | Provide set-aside areas in farmland |
|  | Increase the proportion of natural habitat in the farmed landscape |
|  | Restore species-rich grassland vegetation |
|  | Restore lowland heathland |
|  | Connect areas of natural habitat together |
|  | Reduce tillage |
|  | Increase areas of rough grassland for bumblebee nesting |
|  | Create patches of bare ground for ground-nesting bees |
|  | Provide grass strips at field margins |
|  | Manage hedges to benefit bees |
|  | Increase the use of clover leys on farmland |
|  | Plant dedicated floral resources on farmland |
|  | Sow uncropped arable field margins with an agricultural nectar and pollen mix |
|  | Sow uncropped arable field margins with a native wild flower seed mix |
|  | Leave arable field margins uncropped with natural regeneration |
|  | Increase the diversity of nectar and pollen plants in the landscape |
|  | Reduce intensity of farmland meadow management |
|  | Reduce grazing intensity on pastures |
| **Agricultural chemicals** | Introduce agri-environment schemes generally |
|  | Convert to organic farming |
|  | Restrict certain pesticides |
|  | Reduce pesticide or herbicide use generally |
|  | Reduce fertilizer run-off into margins |
|  | Leave field margins unsprayed within the crop (conservation headlands) |
| **Transport corridors** | Restore species-rich grassland on road verges |
|  | Manage land under power lines for wildlife |
| **Forest management** | Legally protect large native trees |
|  | Re-plant native forest |
|  | Retain dead wood in forest management |
| **Natural processes** | Use prescribed burning to manage habitat and promote flower growth |
| **Non-native species** | Eradicate threatening non-native bees or bee parasites |
|  | Control deployment of non-native hives/nests |
|  | Prevent escape of commercial bumblebees from greenhouses |
|  | Prevent spread of the small hive beetle |
|  | Ensure commercial hives/nests are disease free |
|  | Keep pure breeding populations of native honey bee subspecies |
| **Native predators** | Exclude bumblebee nest predators (such as badgers) from sensitive sites |
| **Artificial nest sites** | Provide artificial nest sites for solitary bees |
|  | Provide artificial nest sites for bumblebees |
| **Captive rearing** | Rear declining bumblebees in captivity |
|  | Reintroduce laboratory-reared queens to the wild |
|  | Reintroduce laboratory-reared bumblebee colonies to the wild |
|  | Translocate bumblebee colonies in nest boxes |
|  | Rear and manage populations of solitary bees |
|  | Translocate solitary bees |
|  | Introduce mated females to small populations to enhance genetic diversity |
| **Awareness** | Enhance bee taxonomy skills through higher education and training |
|  | Provide training to conservationists and land managers on bee ecology and conservation |
|  | Raise awareness amongst the general public through campaigns and public information |
